# Drought-Tolerance Gene Identification Using Genome Comparison and Co-Expression Network Analysis of Chromosome Substitution Lines in Rice

**DOI:** 10.3390/genes11101197

**Published:** 2020-10-14

**Authors:** Chutarat Punchkhon, Kitiporn Plaimas, Teerapong Buaboocha, Jonaliza L. Siangliw, Theerayut Toojinda, Luca Comai, Nuria De Diego, Lukáš Spíchal, Supachitra Chadchawan

**Affiliations:** 1 Program in Biotechnology, Faculty of Science, Chulalongkorn University, Bangkok 10300, Thailand; chutarat_tom@hotmail.com; 2 Center of Excellence in Environment and Plant Physiology, Department of Botany, Faculty of Science, Chulalongkorn University, Bangkok 10300, Thailand; 3 Department of Mathematics and Computer Science, Faculty of Science, Chulalongkorn University, Bangkok 10300, Thailand; kitiporn.p@chula.ac.th; 4 Omics Science and Bioinformatics Center, Faculty of Science, Chulalongkorn University, Bangkok 10300, Thailand; Teerapong.b@chula.ac.th; 5 Molecular Crop Research Unit, Department of Biochemistry, Faculty of Science, Chulalongkorn University, Bangkok 10300, Thailand; 6 National Center for Genetic Engineering and Biotechnology, National Science and Technology Development Agency, 113 Phahonyothin Rd. Khlong Nueng, Khlong Luang, Pathumthani 12120, Thailand; jsiangliw@gmail.com (J.L.S.); theerayut@biotec.or.th (T.T.); 7 Genome Center and Department of Plant Biology, UC Davis Genome Center, UC Davis, Davis, CA 95616, USA; lcomai@ucdavis.edu; 8 Department of Chemical Biology and Genetics, Centre of the Region Haná for Biotechnological and Agricultural Research, Faculty of Science, Palacký University, Šlechtitelů 27, CZ-783 71 Olomouc, Czech Republic; nuria.de@upol.cz (N.D.D.); lukas.spichal@upol.cz (L.S.)

**Keywords:** CSSLs, drought stress, ‘KDML105’ rice, co-expression network

## Abstract

Drought stress limits plant growth and productivity. It triggers many responses by inducing changes in plant morphology and physiology. KDML105 rice is a key rice variety in Thailand and is normally grown in the northeastern part of the country. The chromosome segment substitution lines (CSSLs) were developed by transferring putative drought tolerance loci (QTLs) on chromosome 1, 3, 4, 8, or 9 into the KDML105 rice genome. CSSL104 is a drought-tolerant line with higher net photosynthesis and leaf water potential than KDML105 rice. The analysis of CSSL104 gene regulation identified the loci associated with these traits via gene co-expression network analysis. Most of the predicted genes are involved in the photosynthesis process. These genes are also conserved in *Arabidopsis thaliana*. Seven genes encoding chloroplast proteins were selected for further analysis through characterization of *Arabidopsis* tagged mutants. The response of these mutants to drought stress was analyzed daily for seven days after treatment by scoring green tissue areas via the PlantScreen™ XYZ system. Mutation of these genes affected green areas of the plant and stability index under drought stress, suggesting their involvement in drought tolerance.

## 1. Introduction

Rice (*Oryza sativa* L.) is one of the important cereal crops of the world [1]. In Thailand, rice is the major agricultural export, especially Khao Dawk Mali 105 (KDML105) rice. The cooked kernels of KDML105 rice have a highly prized scent and texture [2]. KDML105 rice is normally grown in the northeast of Thailand, based on rain with limited irrigation [3]. Therefore, it is always affected by drought stress, leading to the reduction in growth and yield.

Drought stress affects plant morphology, physiology, and molecular mechanisms. Upon drought stress, cell turgor pressure is decreased due to low water potential in cells. This causes a decrease in the relative water content, leaf water potential, stomatal conductance, and transpiration rate [4]. Cell expansion and elongation are inhibited, resulting in the reduction of plant height, leaf area, growth, and yield [5]. Photosynthesis is one of the important physiological mechanisms affected by drought stress. The decrease in leaf expansion, leaf area, and stomatal conductance limits CO_2_ uptake [6]. The photosynthetic pigments (chlorophyll *a*, chlorophyll *b*, and carotenoids) can also be damaged by drought stress, resulting in their degradation and decreased light absorption and maximum photosynthetic rate [7]. During drought stress, phosphoenolpyruvate carboxylase, nicotinamide adenine dinucleotide phosphate-malic enzyme, Rubisco, fructose-1,6-bisphosphatase, and pyruvate orthophosphate dikinase activities are decreased, which can reduce the photosynthetic and electron transport rate [8]. The physiological responses to drought tolerance include osmotic adjustment, osmoprotection, antioxidation, scavenging defense, and photorespiration [9,10].

Kanjoo et al. [11] developed chromosome segment substitution lines (CSSLs) in the background of variety KDML105. CT9993, a variety with a good rooting system, and IR62266, a variety with high osmotic adjustment ability, were hybridized, and their F1 was used to generate double haploid lines. The double haploid lines were evaluated for yield, yield components, and morpho-physiological characters under drought-stress conditions, defining drought-tolerant quantitative trait loci (DT-QTLs) on chromosomes 1, 3, 4, 8 and 9. The doubled haploid line DH212 carries CT9993 alleles on in all chromosomes, while DH103 has IR62266 alleles on chromosome 8. These lines were selected as donor lines for CSSL development. Repeated crossing to KDML105 resulted in CSSLs with the putative drought-tolerant genes from chromosome 8, donated by DH103, and the CSSLs with the DT-QTL from chromosome, 1, 3, 4 and 9, donated by DH212 [3].

CSSL104 is a drought-tolerant KDML105 CSSL carrying the chromosome 8 introgression from inbred DH103 [11]. Compared to KDML105, CSSL104 had higher relative water content, higher chlorophyll fluorescence (F_v_/F_m_), and lower leaf drying score under 50% field capacity drought-stress conditions [12]. This implied that the region introgressed from DH103 carried the putative drought-tolerant genes.

To investigate the mechanisms affected by the introgression of DH103 genes, the physiological responses to drought stress of CSSL104 were evaluated relative to KDML105 and the DH103 donor line. Then, drought-tolerance genes were predicted based on genomic sequence comparison and co-expression network analysis. Finally, the putative drought-tolerance genes were validated using the corresponding *Arabidopsis* mutants. This study will be beneficial to the future development of drought-tolerant rice.

## 2. Materials and Methods

### 2.1. Plant Materials

We used CSSLs with the genetic background of KDML105 rice and containing a putative drought-tolerance segment of chromosome 8 from DH103 between markers RM5353 and RM3480 [11]. These are CSSL97, CSSL104, CSSL106, CSSL107, and parental lines (KDML105 and DH103). They were used to study drought-stress responses. All rice seeds were provided by the Innovative Plant Biotechnology and Precision Agriculture Research Team (APBT) at the National Center for Genetic Engineering and Biotechnology (BIOTEC), Thailand. 

### 2.2. Evaluation of Physiological Responses at Vegetative Stage under Drought-Stress Conditions

#### 2.2.1. Rice Growth Condition

KDML105 rice, DH103, and CSSL seeds were incubated at 60 °C for 48 h before planting. The seeds were then germinated by soaking in distilled water for seven days in plastic cups. Rice seedlings were transferred to a plastic tray and continuously grown in WP nutrient solution [13]. Twenty-eight days after germination, rice plants were drought-stressed for three days by the addition of 10% polyethylene glycol 6000 (PEG6000). This condition was previously shown to cause drought stress in rice [14,15]. In order to induce the stronger drought-stress condition, after treatment with WP nutrient solution with 10% PEG6000 for three days, the solution was then changed to WP nutrient solution with 15% PEG. Plants grown in WP nutrient solution without PEG6000 were used as controls. A complete randomized design (CRD) with four replicates was used for physiological evaluation in each parameter.

#### 2.2.2. Net Photosynthesis Rate and Leaf Water Status Detection

The net photosynthesis rate (Pn) of twenty-eight-day-old rice plants was determined with a LI-6400 XT portable photosynthesis system (LI-COR, Lincoln, NE, USA). The measurement was taken at the middle part of the youngest fully expanded leaves between 9 am and 2 pm, with the following conditions: the molar flow of air per unit leaf area was 500 mmol l^−1^ m^−2^ s^−1^, the photosynthetically active radiation (PAR) at the leaf surface was 1200 μmol m^−2^ s^−1^, the leaf temperature ranged from 30.0 to 37.0 °C, with a CO_2_ concentration of 380.0 mol mol^−1^. The leaf water potential (LWP) was measured in the youngest fully expanded leaves using plant water status console model 3005 (Soilmoisture, Goleta, CA, USA).

### 2.3. Identification of Drought-Tolerance Gene

#### 2.3.1. Whole Genome Sequencing

The aboveground parts of KDML105, DH103, and CSSL104 rice plants were collected at fourteen days after germination. Rice genomic DNA was extracted using a Genomic DNA Mini Kit, ‘Plant’ (Geneaid, New Taipei City, Taiwan). Genomic DNA libraries were prepared for sequencing by using an Illumina genome analyzer (Illumina, San Diego, CA, USA) with Illumina HiSeq3000’s protocol. For genome analyses, the sequence reads were classified into specific categories using the pipeline developed by Missirian et al. [16]. The rice genomic sequence from the Rice Genome Annotation Project database [17] was used as a reference genome [18] to map the sequence reads. The raw reads were submitted to GenBank at the NCBI under BioProject no. PRJNA659381. Bioinformatic tools were used to compare the genome of CSSL104 with the KDML105 rice genome to identify loci containing different single nucleotide polymorphisms (SNPs). These loci may contribute to the drought-tolerance phenotypes of CSSL104. The genome comparison first started by discarding all SNPs shared by both CSSL104 and KDML105. The remaining differential SNPs were counted within a sliding window of 5000 background nucleotides. To visualize the chromosome plots with the marks of different SNPs’ loci, the window region containing more than 100 SNPs in CSSL104 with different nucleotides from KDML105 was marked as a blue line on the chromosome plots. The analysis of the locations of SNPs in the candidate genes was analyzed.

#### 2.3.2. Gene Co-Expression Network Analysis

The rice loci containing the different SNPs were used for a gene co-expression network analysis. To predict the important loci involved in drought tolerance, a rice oligonucleotide array database was used with abiotic stress-induced gene expression data with a correlation coefficient cut-off of 0.95 [19]. The predicted loci were searched for gene ontology and expression patterns from the rice expression database [20].

### 2.4. Identification of Drought-Tolerance Gene Function in Arabidopsis

#### 2.4.1. *Arabidopsis* Homologous Gene

The best candidate genes were used to search for the homologous gene in *Arabidopsis* from the Rice Genome Annotation Project database [17] and The *Arabidopsis* Information Resource [19]. *Arabidopsis* mutant lines with T-DNA insertion in the selected gene were ordered from the Arabidopsis Biological Resource Center (ABRC). The *Arabidopsis* seeds were screened for homozygous mutant lines via specific primers, LP and RP, to the gene of interest; the LB primer was used for a specific T-DNA region.

#### 2.4.2. *Arabidopsis* Growth Condition

Four days after germination, *Arabidopsis thaliana* ecotype Col-0 seeds and seven mutant lines [21] including *at1g74880*, *at5g54270*, *at3g63190*, *at4g11960*, *at4g22890*, *at2g27680*, and *at4g34830* were sowed and transferred to 48-well plates, containing Murashige and Skoog (MS) agar media for normal conditions, MS agar media supplemented with 75 mM mannitol for mild drought stress, or MS agar media supplemented with 150 mM mannitol for severe drought stress. The plants were then grown in a growth chamber at 22 ℃/20 ℃, 16/8 h light/dark cycle, 120 µmol photons of PAR m^−2^·s^−1^, and 60% humidity. RGB imaging was used to collect the green area of plants twice a day via a PlantScreenTM XYZ system (Photon Systems Instruments, Drásov, Czech Republic) [22]. 

### 2.5. Statistical Analysis

Analysis of variance (ANOVA) and Duncan’s Multiple Range Test (DMRT) were used for data analysis by SPSS Statistics program version 22 (IBM, Armonk, NY, USA). The images from the PlantScreenTM XYZ system were analyzed using MATLAB (R2015; MathWorks, Inc., Natick, MA, USA), and the data were analyzed by independent *t*-tests using SPSS.

## 3. Results

### 3.1. Evaluation of Physiological Responses of CSSLs of KDML105 under Drought-Stress Conditions

**Figure 1:**
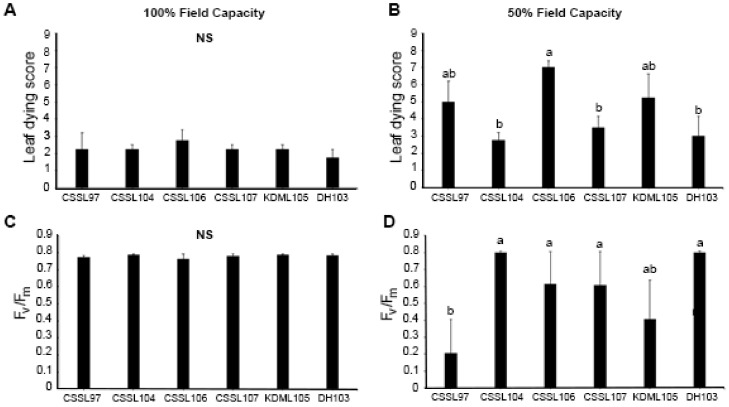
Response to drought stress in chromosome segment substitution lines (CSSLs) and parents. CSSLs, CSSL97, CSSL104, CSSL106, and CSSL107, and their parental lines KDML105 and DH103, were compared for leaf death (leaf dying score) and photosystem II efficiency (F_v_/F_m_) under (**A**,**C**) normal (100% field capacity) and (**B**,**D**) drought-stress (50% field capacity) conditions. The mean + 1 standard error (SE) was derived from four replicates. Means with a different lowercase letter above them are significantly different (*p* < 0.05). NS demonstrates no significant difference among lines.

Selected CSSLs, namely CSSL97, CSSL104, CSSL106, and CSSL107, were evaluated for drought tolerance by growing the seedlings in soil with 100% or 50% field capacity. In normal growth conditions (100% field capacity), all of the lines were similar (Figure 1A,C), but they differed under 50% field capacity (Figure 1B,D). CSSL104 displayed the most drought-tolerant phenotype, with the lowest leaf dying score and the highest photosystem II (PSII) efficiency (F_v_/F_m_). This was similar to the performance of DH103 (the drought-tolerant parental line). The highest leaf-death score was detected in CSSL106, while CSSL97 had the lowest PSII efficiency under drought stress. These data suggest that CSSL104 is the most drought-tolerant line, while CSSL97 and CSSL106 are the most susceptible. Therefore, CSSL104 was selected for further characterization.

CSSL104 and its parental lines, KDML105 and DH103 rice, were grown in nutrient solutions corresponding to normal growth conditions and in a solution supplemented with 15% PEG for the drought-stress treatment, which caused about a 50–180% reduction in leaf water potential (Table 1). After nine days of drought stress, we measured the highest reduction of leaf water potential for all lines. Under these conditions, CSSL104 had the lowest leaf water potential at −7.75 MPa, which was about threefold lower than the LWP of the plants grown in normal conditions. The parental lines, KDML105 and DH103, had about a twofold reduction in LWP. Drought stress reduced all parameters of photosynthesis, including net photosynthesis rate, stomatal conductance, transpiration rate, intercellular CO_2_ concentration, ΦPSII, and electron transport rate in all lines. However, after six days under drought stress, CSSL104 had a significantly higher photosynthetic rate than the KDML105 parent. In addition, CSSL104 had a greater tendency than KDML105 toward higher values for all photosynthetic parameters after nine days of drought treatment (Table 1).

### 3.2. Whole Genome Sequence Comparison between CSSL104 and KDML10’ and Co-Expression Network Analysis Revealed that Major Hub Genes Have a Role in Photosynthesis

**Figure 2:**
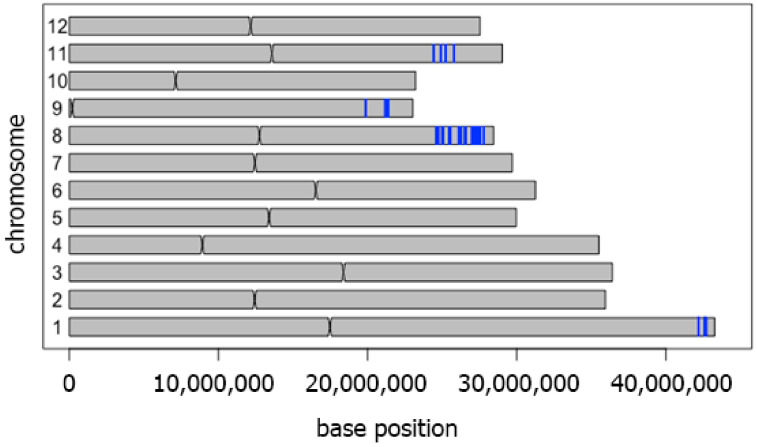
Genetic regions introgressed into the KDML105 genome. Single nucleotide polymorphisms (SNPs) between CSSL104 and KDML105 rice. The blue lines show 100 SNPs within 5000 background nucleotides. All loci containing SNPs were subjected to a co-expression network analysis. The results, shown in Figure 3A, revealed 18 major nodes with a high connection to other genes. The gene ontology of these 18 genes is listed in Table 2. The map position of these genes is shown in Figure 3B. Based on quantitative trait loci (QTL) data from the Qtaro database [23], six loci are located in QTL regions for drought-stress tolerance on chromosomes 1, 3, and 8 (Figure 3B). The high-density SNP on chromosome 1 was consistent with the location of the drought-tolerant (DT)-QTL, which is flanked by markers RZ14 and R117. In this QTL, co-expression network analysis identified two genes, *LOC_Os01g72800* and *LOC_Os01g72950*, as the major nodes. Chromosome 3 did not display high-density SNPs. On this chromosome, *LOC_Os03g02590* and *LOC_Os03g03910* were located in two drought-tolerance QTLs mapped between markers RM7332, RM545, and RG104, RZ329. Another node gene, *LOC_Os03g52460,* is located between markers C136 and R1618, corresponding to another drought-tolerance QTL. Chromosome 8 displays several major nodes: *LOC_Os08g16570* is located between markers RM72 and RM331, while *LOC_Os08g41040* and *LOC_Os08g41460* are located between RM5353 and RM 3480. This region on chromosome 8 also displayed high-density SNPs between CSSL104 and KDML105 (Figure 2 and Figure 3B).

We compared whole genome sequences of CSSL104 and its drought susceptible parental line KDML105 to define the genes responsible for drought tolerance in CSSL104. A total of 101,950 SNPs located on 3440 genes were detected. The regions with a high density of SNPs were on chromosomes 1, 8, 9, and 11 (Figure 2). 

The eighteen rice genes reported here have homologs in *Arabidopsis* for which tagged mutants are available (Table 2). Nine of them (*CPFTSY*, *NDH-O*, *SOQ1*, *LHCB3*, *RRF*, *PGRL1B*, *HCF244*, *NAD(P)-linked oxidoreductase,* and *MRL1*) were annotated to be involved in the photosynthesis process [24,25,26,27,28,29,30,31,32]. Moreover, the homolog of *LOC_Os11g43600* is *CPRF1,* an *Arabidopsis* gene required for chloroplast development [33]. These findings suggest that these rice genes are involved in photosynthesis adaptation during drought stress.

Therefore, we obtained seven homozygous, T-DNA tagged *Arabidopsis* mutant lines corresponding to *ndhO* (*at1g74880*), *lhcb3* (*at5g54270*), *rrf* (*at3g63190*), *pgrl1b* (*at4g11960*), *pgrl1a* (*at4g22890*), *at2g27680*, and *mrl1* (*at4g34830*). These lines were drought stressed by growing them in MS medium supplemented with 0 mM, 75 mM, or 150 mM mannitol. Their growth response was assessed by measuring the green pixel area per plant and compared to the wild type (WT).

Under normal conditions, all mutant lines displayed a significantly lower number of green pixels than WT, suggesting lower growth than WT (Figure 4A). Both drought-stress treatments decreased growth in all lines, with the 150 mM causing the more severe reduction. At 75 mM mannitol, *pgrl1b* had a significantly lower number of green pixels than WT, while *pgrl1a* showed similar growth to WT. Other mutant lines showed better growth than WT (Figure 4B). Under the severe drought-stress conditions induced with 150 mM mannitol, the growth of *lhcb3*, *at2g27680*, *mrl1,* and WT were similar. Mutants *pgrl1b* and *pgrl1a* had a significantly lower growth than WT, while *rrf* had a lower growth at the beginning of the treatment but displayed better growth than WT after 5 days of the treatment. However, similar growth between WT and *rrf* was found after seven days of drought stress. Among the mutant lines, *ndhO* was the only mutant line that had significantly better growth than WT under severe drought stress (Figure 4C).

The stability indexes of *Arabidopsis* mutants and WT were calculated to compare drought tolerance after six days of drought stress. After the intermediate drought stress (75 mM mannitol), all mutant lines except *pgrl1a* displayed significantly higher stability than WT, suggesting the contribution of *NDH-0*, *LHCB3*, *RRF*, *PGRL1b, at2g27680,* and *MRL1* to drought-tolerance adaptation (Figure 5A). Under severe drought (150 mM mannitol), significantly higher stability than WTs was detected for the *ndh-o, rrf*, *pgrl1b, at2g27680,* and *mrl1* mutants (Figure 5B). The *rrf* mutant line displayed the highest stability under both intermediate and severe drought-stress conditions.

## 4. Discussion

We investigated the effect of a drought-stress QTL introgressed into the elite rice line KDML105. Using leaf water potential under drought stress, we found that introgression line CSSL104 manifested drought tolerance similar to that of the parent line DH103. Both tended to have a better ability to maintain water status in the first fully expanded leaves. After six days of drought stress, both lines had about a 22% higher leaf water potential than the KDML105 parent. Drought stress limits water uptake from the rice root and reduces water availability in the cells, which is critical for survival under drought stress. Water depletion can compromise photosynthesis and cell growth [34], and three main maintenance mechanisms are used by plants to offset water loss: leaf rolling, stomatal closure, and osmoregulation [35,36]. Evidence for the drought tolerance of CSSL104 was also provided by the lower leaf-death score and higher F_v_/F_m_ displayed by CSSL104 compared to KDML105 (Figure 1).

Drought stress resulted in decreased stomatal conductance in all lines (KDML105, DH103, and CSSL104; Table 1). This was a water-preservation mechanism that also resulted in a decline in the net photosynthesis rate. After three days of drought stress, the photosynthesis parameters, net photosynthesis rate, stomatal conductance, transpiration rate, ΦPSII, electron transport rate, intercellular CO_2_ concentration, and F_v_’/F_m_’, were similar among all lines. After six days under drought stress, the net photosynthesis rate of DH103 and CSSL104 were about twofold higher than the net photosynthesis rate of KDML105. In comparison with normal plants, KDML105 rice had a nearly 70% reduction in photosynthesis rate, while DH103 and CSSL104 had only a 46 and 44% reduction, respectively. Interestingly, the ΦPSII and electron transport rate of all lines were similar, while the stomatal conductance of KDML105 was 58% lower than DH103. These findings suggest that stomatal closure could be one of the major factors contributing to the decline in the net photosynthesis rate of KDML105. Although the stomatal conductance of CSSL104 was lower than DH103 by 42%, CSSL104 could maintain a net photosynthesis rate (Table 1). These indicated that this CSSL is better adapted than its drought-tolerant parental line. It is possible that a KDML105 locus contributed to maintenance of the photosynthesis process through an epistatic interaction with the introgressed DH103 region.

**Figure 3:**
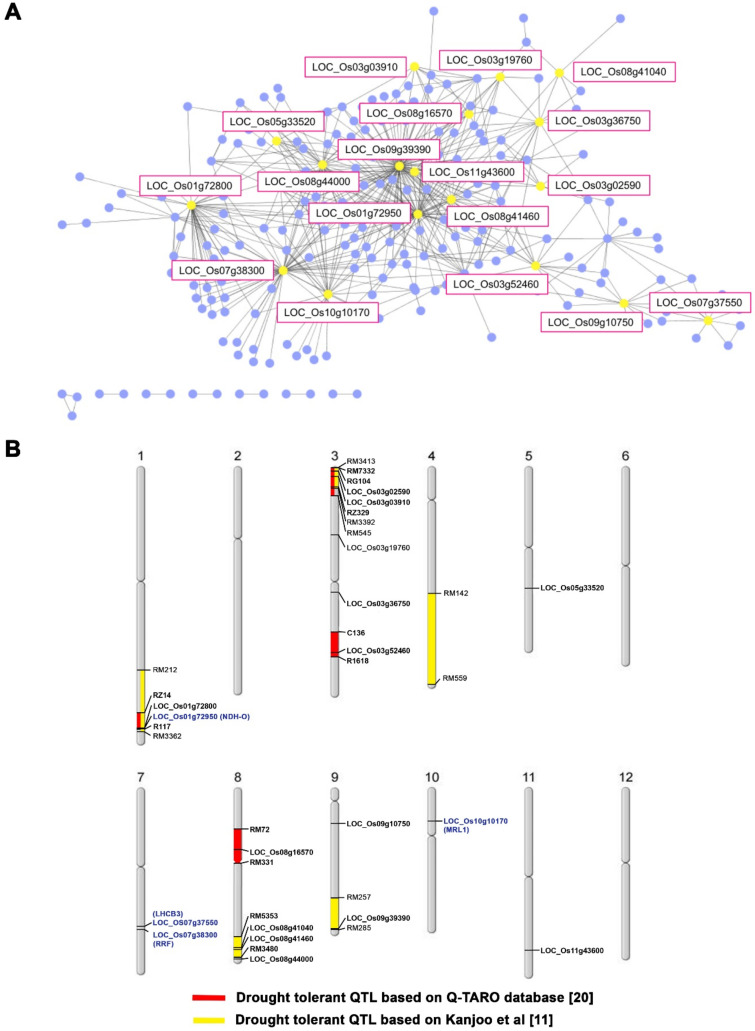
Candidate genes for drought tolerance. Gene co-expression network was analyzed by using the Rice Oligonucleotide Array Database [19], showing the major node genes with yellow dots (**A**), while DT-QTLs from the Q-TARO database [23] and Kanjoo et al. [11] are shown in red and yellow boxes on the chromosome, respectively. (**B**) Loci written in blue letters indicate the proposed drought-tolerance loci based on this study.

Using whole genome sequence comparison and co-expression network analysis, we characterized the molecular fingerprint of the introgression. The first detected DNA segments introgressed, while the second identified genes connected with the drought response. During this stress, 18 genes were highly co-expressed with other genes (Figure 3A and Table 2). Nine of them (*CPFTSY*, *NDH-O*, *SOQ1*, *LHCB3*, *RRF*, *PGRL1*, *HCF244*, *NAD(P)-linked oxidoreductase,* and *MRL1*) are involved in the photosynthesis process, and CPRF1 is essential for chloroplast development. These findings indicate that the drought-adaptation QTL affects photosynthetic genes whose modulation maintains the net photosynthesis rate of CSSL104.

**Figure 4:**
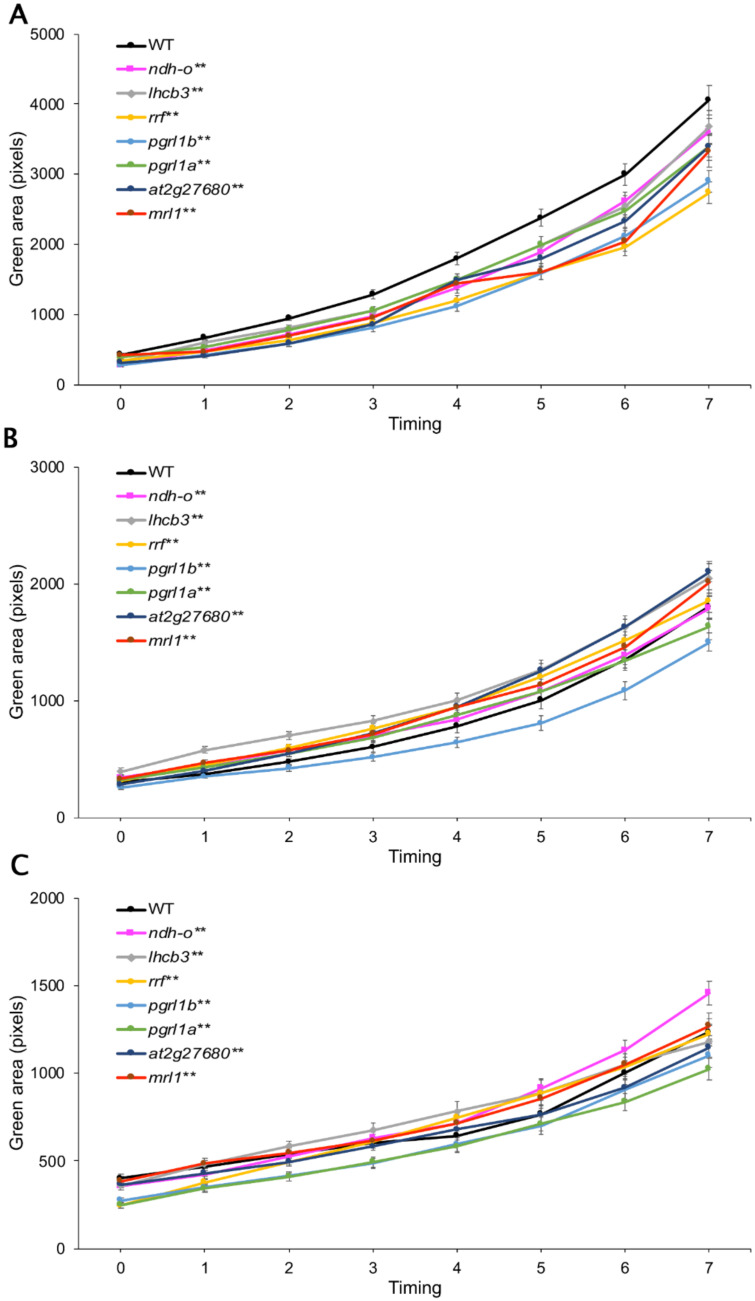
Growth response of seven mutant *Arabidopsis* lines to drought stress. Comparison of growth (green pixels per plant) of wild type (WT) and the T-DNA insertion mutant lines *ndhO* (*at1g74880*), *lhcb3* (*at5g54270*), *rrf* (*at3g63190*), *pgrl1b* (*at4g11960*), *pgrl1a* (*at4g22890*), *at2g27680*, and *mrl1*(*at4g34830*) grown in (**A**) normal Murashige and Skoog (MS) medium, (**B**) under intermediate drought stress (MS medium supplemented with 75 mM mannitol), and (**C**) under severe drought stress (MS medium supplemented with 150 mM mannitol). Statistical analysis was by *t*-test. * and ** above the name of the mutant line represent significant difference (*p* < 0.05) and highly significant difference (*p* < 0.01) between WT and mutant, respectively.

The function of the identified genes is as follows. CPFTSY (chloroplast FtsY, i.e., chloroplast signal-recognition particle) is required for light-harvesting chlorophyll *a*/*b*-binding protein (LHCP) integration into thylakoids [25], and NDH-O is the subunit required for the NADH dehydrogenase-like (NDH) complex assembly that functions in cyclic electron flow [25,37]. SOQ1 is required to maintain light harvesting efficiency especially during nonphotochemical quenching (NPQ) recovery [26]. Light-harvesting chlorophyll (LHC) functions as a light receptor to capture light energy and deliver it to photosystems. The *Lhcb3* gene product regulates the rate of state transition by changing the excitation energy transfer and charge separation [38]. RRF is a ribosome recycling factor in chloroplast [39]. *RRF* is required to maintain photosystem II efficiency (F_v_/F_m_) and proper stacking of the internal membranes of chloroplast. Loss of these functions led to a lower growth rate for the *rrf* mutant compared to WT [28], which is consistent with the phenotype documented in this study (Figure 4A). *PGRL1A* (*AT4G22890*) and *PGRL1B* (*AT4G11960*) are paralogous genes whose products switch linear electron flow to cyclic electron flow. PGRL1 is the elusive ferredoxin-plastoquinone reductase (FQR) [29].

**Figure 5:**
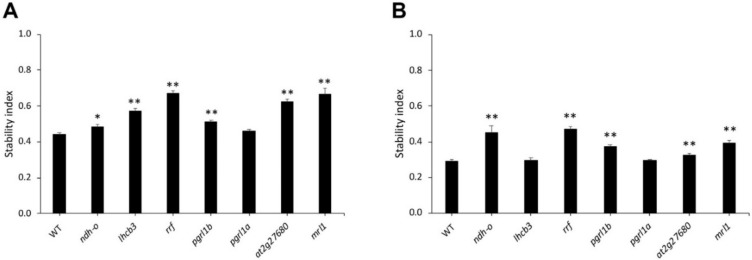
Stability during drought stress. The stability index, which is the ratio between the values from stressed plants and normal growth plants, is shown in (**A**) 75 mM mannitol and (**B**) 150 mM. This figure represents the mean ± SE of WT and mutant lines. The * shows a significant difference at *p* values ≤ 0.05 and ** indicates *p* value ≤ 0.01.

During drought stress, plants shift the electron transfer route from linear to cyclic to balance the energy flow from light reaction to Calvin cycle and photorespiration [37,40,41]; this change is due to CO_2_ limitations caused by stomatal closure. It was recently shown that tomato with co-silencing of the *PGR5*/*PGRL1A* gene was more susceptible to cold stress [42]. This is consistent with our finding that the *pgrl1* mutant had a significantly lower growth rate than WT in both intermediate and severe drought stress (Figure 5B,C). HCF244 is required for the biogenesis of photosystem II (PSII), and specifically for the synthesis of the reaction center proteins [30,31,32]. The NAD(P)-linked oxidoreductase gene is involved in redox reactions, but the details are still unclear. *MRL1* is the only gene in this study whose product is involved in the Calvin cycle. It is involved either in the processing or in the stabilization of the large subunit (LS) of *RuBisCO* transcripts [43].

Not surprisingly, impairment of these photosynthesis genes significantly reduced growth under normal conditions (Figure 4A). Moreover, we could not obtain homozygous lines of the *soq1*, *hcf244*, and *cprf1* mutation, probably because the homozygous mutants are lethal. However, under drought-stress conditions, some of the mutants in the genes involved in the light reaction process (*ndhO* (*at1g74880*), *lhcb3* (*at5g54270*), *rrf* (*at3g63190*), and *at2g27680* mutants) displayed significantly higher growth than WT. These responses suggested that the decrease in light energy harvest during drought stress could prevent damage to chloroplasts and prolong survival of photosynthetic tissues. The *mrl1* mutant was expected to lack of the ability to stabilize the *rbcL* mRNA, which could result in a decline in Calvin cycle activity. The higher stability index of the *mrl1* mutant line under intermediate and severe drought stress indicated that a slower rate of the Calvin cycle may help plants cope with drought stress.

Based on the growth phenotype under drought stress, the *ndhO*, *lhcb3*, *rrf*, and *mrl1* mutants showed a higher growth rate than WT (Figure 4B,C). The homologs in rice of these genes are *LOC_Os01g72800*, *LOC_Os07g37550*, *LOC_Os07g38300*, and *LOC_Os10g10170*, respectively. Therefore, we would like to propose that these genes contribute to drought-tolerance regulation in rice by mediating adaptation in the photosynthesis process. *LOC_Os01g72800* is located in the previously reported drought-tolerance QTL between RZ14 and R117 [21], while the other three genes are not. Collectively, our results indicate that the combination of SNP analysis with co-expression network analysis is a suitable method for drought-tolerance gene prediction. This approach will help future exploration to identify the candidate genes for abiotic stress tolerance.

## 5. Conclusions

The KDML105 chromosome substitution line CSSL104 displayed a drought-tolerance phenotype based on photosynthetic maintenance ability. Identification of SNPs between KDML105 and the tolerant CSSL, together with co-expression network analysis, predicted 18 candidate drought-tolerance genes—ten of which were involved in photosynthesis or chloroplast development. Seven of them were selected for the characterization by using *Arabidopsis* mutant lines for the homologous genes. Four out of seven mutants showed a higher growth rate than WT under drought stress. Therefore, *LOC_Os01g72800*, *LOC_Os07g37550*, *LOC_Os07g38300*, and *LOC_Os10g10170* are proposed to be the drought-tolerance genes in CSSL104 rice.

## Figures and Tables

**Table 1:** Photosynthetic performance of CSSL104 and parent lines. Net photosynthesis rate, transpiration rate, stomatal conductance, ΦPSII, electron transport rate, intercellular CO_2_ concentration, F_v_’/F_m_’, relative chlorophyll content (determined by portable chlorophyll meter SPAD-501) and leaf water potential of rice at vegetative stage in normal and drought stress conditions. ANOVA and Duncan’s Multiple Range Test (DMRT) were used for statistical analysis. The data show mean ± SE. Different superscript letters show the significant difference among lines at *p* value ≤ 0.05.

Conditions	Normal (0% PEG)				Drought Stress (15% PEG)			
Timing (Days after Stress)	0	3	6	9	0	3	6	9
Net photosynthesis rate (μmol·m^−2^·s^−1^)								
KDML105	19.44 ± 4.46	14.50 ± 0.81	17.85 ± 1.85	20.17 ± 0.88	19.44 ± 4.46	10.30 ± 1.74	5.51 ± 0.42 ^b^	8.64 ± 1.11
DH103	14.47 ± 2.31	17.45 ± 1.21	18.92 ± 1.53	21.35 ± 2.02	14.47 ± 2.31	10.67 ± 0.38	10.20 ± 1.23 ^a^	12.35 ± 0.59
CSSL104	15.12 ± 1.28	16.74 ± 1.10	18.47 ± 1.61	15.76 ± 0.87	15.12 ± 1.28	11.31 ± 1.88	10.31 ± 1.33 ^a^	9.38 ± 1.79
Transpiration rate (mmol·m^−2^·s^−1^)								
KDML105	5.26 ± 0.56	4.86 ± 0.44	0.23 ± 0.01	5.10 ± 0.39 ^b^	5.26 ± 0.56	2.42 ± 0.14	0.18 ± 0.02	1.87 ± 0.26 ^b^
DH103	4.99 ±0.48	5.87 ± 0.12	0.26 ± 0.02	6.77 ± 0.71 ^a^	4.99 ±0.48	2.65 ± 0.24	0.17 ± 0.02	3.33 ± 0.10 ^a^
CSSL104	4.54 ± 0.28	4.99 ± 0.34	0.25 ± 0.01	4.48 ± 0.27 ^b^	4.54 ± 0.28	2.57 ± 0.32	0.17 ± 0.02	2.16 ± 0.34 ^b^
Stomatal conductance (mmol·m^−2^·s^−1^)								
KDML105	0.35 ± 0.09	0.32 ± 0.05	0.32 ± 0.06 ^b^	0.33 ± 0.04 ^b^	0.35 ± 0.09	0.12 ± 0.01	0.08 ± 0.01	0.09 ± 0.01 ^b^
DH103	0.43 ± 0.05	0.44 ± 0.02	0.44 ± 0.03 ^a^	0.53 ± 0.07 ^a^	0.43 ± 0.05	0.12 ± 0.01	0.19 ± 0.04	0.17 ± 0.01 ^a^
CSSL104	0.36 ± 0.03	0.33 ± 0.06	0.33 ± 0.06 ^b^	0.28 ± 0.02 ^b^	0.36 ± 0.03	0.15 ± 0.02	0.11 ± 0.02	0.10 ± 0.02 ^b^
ΦPSII								
KDML105	0.25 ± 0.00	0.23 ± 0.01	0.23 ± 0.01	0.23 ± 0.01 ^b^	0.25 ± 0.00	0.20 ± 0.01	0.18 ± 0.02	0.15 ± 0.01
DH103	0.22 ± 0.02	0.25 ± 0.02	0.26 ± 0.02	0.29 ± 0.01 ^a^	0.22 ± 0.02	0.21 ± 0.03	0.17 ± 0.02	0.18 ± 0.02
CSSL104	0.24 ± 0.01	0.21 ± 0.01	0.25 ± 0.01	0.22 ± 0.00 ^b^	0.24 ± 0.01	0.21 ± 0.01	0.17 ± 0.02	0.17 ± 0.01
Electron transport rate								
KDML105	163.02 ± 2.37	149.08 ± 9.83	153.51 ± 4.96	152.38 ± 3.91 ^b^	163.02 ± 2.37	130.73 ± 4.36	117.25 ± 10.71	100.65 ± 8.13
DH103	143.72 ± 10.16	163.29 ± 11.08	168.02 ± 11.52	188.68 ± 4.99 ^a^	143.72 ± 10.16	139.94 ± 16.65	110.58 ± 11.43	118.43 ± 10.95
CSSL104	155.86 ± 6.09	141.86 ± 7.08	161.10 ± 6.45	144.28 ± 2.79 ^b^	155.86 ± 6.09	133.31 ± 11.19	113.12 ± 12.52	112.18 ± 8.52
Intercellular CO_2_ concentration (μmol·mol^−1^)								
KDML105	327.27 ± 5.37	305.04 ± 11.48	282.47 ± 14.96 ^b^	282.98 ± 10.67	327.27 ± 5.37	255.39 ± 18.30	269.74 ± 18.42	215.42 ± 21.55 ^a^
DH103	330.97 ± 5.82	318.58 ± 3.45	312.79 ± 8.84 ^a^	313.36 ± 8.12	330.97 ± 5.82	260.64 ± 19.39	286.68 ± 18.62	265.87 ± 8.18 ^b^
CSSL104	317.29 ± 2.12	294.48 ± 11.18	281.92 ± 13.68 ^b^	289.50 ± 9.46	317.29 ± 2.12	241.03 ± 14.35	227.89 ± 18.71	237.02 ± 13.81 ^ab^
F_v_’/F_m_’								
KDML105	0.54 ± 0.01 ^b^	0.53 ± 0.01	0.50 ± 0.01	0.50 ± 0.02	0.54 ± 0.01 ^b^	0.50 ± 0.01	0.48 ± 0.01	0.45 ± 0.02
DH103	0.63 ± 0.02 ^a^	0.60 ± 0.03	0.55 ± 0.02	0.52 ± 0.01	0.63 ± 0.02 ^a^	0.53 ± 0.04	0.53 ± 0.08	0.47 ± 0.03
CSSL104	0.53 ± 0.01 ^b^	0.54 ± 0.01	0.53 ± 0.03	0.52 ± 0.01	0.53 ± 0.01 ^b^	0.52 ± 0.03	0.48 ± 0.01	0.43 ± 0.02
Relative chlorophyll content								
KDML105	35.93 ± 0.41	36.63 ± 0.95	36.40 ± 0.81	38.30 ± 1.05	35.93 ± 0.41	37.58 ± 0.40	37.10 ± 0.57	34.20 ± 1.02
DH103	34.43 ± 0.43	35.78 ± 0.21	36.55 ± 0.34	38.15 ± 0.95	34.43 ± 0.43	33.25 ± 1.12	32.58 ± 2.18	34.38 ± 0.97
CSSL104	34.90 ± 0.65	35.70 ± 0.39	38.65 ± 1.70	39.00 ± 0.38	34.90 ± 0.65	35.93 ± 1.06	36.25 ± 0.71	34.48 ± 1.21
Leaf water potential (MPa)								
KDML105	−1.20 ± 0.14	−2.70 ± 0.77	−5.15 ± 1.08	−2.90 ± 0.26	−1.60 ± 0.08	−4.05 ± 0.48	−9.00 ± 1.00	−6.85 ± 0.43
DH103	−1.35 ± 0.10	−2.60 ± 0.54	−3.90 ± 0.33	−3.25 ± 0.10	−2.05 ± 0.32	−4.60 ± 0.50	−7.00 ± 0.38	−6.95 ± 1.03
CSSL104	−1.20 ± 0.14	−1.95 ± 0.15	−4.20 ± 1.29	−2.70 ± 0.13	−1.65 ± 0.05	−4.08 ± 0.31	−7.10 ± 1.04	−7.75 ± 1.50

**Table 2:** Rice gene candidates for drought tolerance, their *Arabidopsis* homologs, and the inferred function of the genes in rice.

Rice Locus ID	*Arabidopsis* Locus ID	Gene Description	Mutant Stock	Homozygous	Involved in Photosynthesis
LOC_Os01g72800	AT2G45770	Chloroplast signal recognition particle (SRP) receptor homolog, alpha subunit CPFTSY. Required for light-harvesting chlorophyll *a/b*-binding protein (LHCP) integration into isolated thylakoids.	SALK_070410C	✓	✓
LOC_Os01g72950	AT1G74880	NDH-O, encoding subunit NDH-O of NAD(P)H: plastoquinone dehydrogenase complex (Ndh complex) present in the thylakoid membrane of chloroplasts. This subunit is thought to be required for Ndh complex assembly.	SALK_097351C	✓	✓
LOC_Os03g02590	AT1G01820	PEROXIN11C, member of the peroxin11 (PEX11) gene family, integral to peroxisome membrane, controls peroxisome proliferation	SALK_057358C	✓	
LOC_Os03g03910	AT4G35090	CAT2	SALK_076998	✓	
LOC_Os03g19760	AT1G56500	SOQ1 (Suppressor of quenching 1) prevents the formation of a slowly reversible form of antenna quenching, thereby maintaining the efficiency of light harvesting.	SALK_097577		✓
LOC_Os03g36750	AT3G48420	Haloacid dehalogenase-like hydrolase (HAD) superfamily protein	SALK_025204	✓	
LOC_Os03g52460	AT5G19220	APL1, the large subunit of ADP-glucose pyrophosphorylase, which catalyzes the first and rate-limiting step in starch biosynthesis.	CS478981	✓	
LOC_Os05g33520	AT2G48070	RPH1 is a chloroplast protein RPH1 (resistance to Phytophthora 1) involved in immune response to *Phytophthora brassicae*	SALK_102558C	✓	
LOC_Os07g37550	AT5G54270	LHCB3 is a component of the main light-harvesting chlorophyll a/b-protein complex of Photosystem II (LHC II)	SALK_020314C	✓	✓
LOC_Os07g38300	AT3G63190	RRF, encoding a chloroplast ribosome recycling factor homolog.	SALK_015954C	✓	✓
LOC_Os08g16570	AT1G16080	Nuclear protein	SALK_007790C	✓	
LOC_Os08g41040	AT4G31115	DUF1997 family protein	SALK_010690C	✓	
LOC_Os08g41460	AT4G11960	PGRL1B—a transmembrane protein present in thylakoids. Plants lacking PGRL1 show perturbation of cyclic electron flow.	SALK_059238C	✓	✓
	AT4G22890		SALK_133856C	✓	✓
LOC_Os09g10750	AT2G42220	Rhodanese/cell cycle control phosphatase superfamily protein	SALK_045769	✓	
LOC_Os09g39390	AT2G27680	NAD(P)-linked oxidoreductase superfamily protein	SALK_073120C	✓	✓
LOC_Os10g10170	AT4G34830	MRL1 (a conserved pentatricopeptide repeat protein) required for stabilization of *rbcL* mRNA	SALK_060806C	✓	✓
LOC_Os11g43600	AT3G62910	Chloroplast ribosome release factor 1, CPRF1, encoding a plastid-localized ribosome release factor 1 that is essential in chloroplast development	SALK_117765C		

## References

[1] Saikumar S., Gouda P.K., Saiharini A., Varma C.M.K., Vineesha G., Padmavathi G., Shenoy V.V. (2014). Major QTL for enhancing rice grain yield under lowland reproductive drought stress identify using *O. sativa*/*O. glaberrima* introgression line. Field Crops Res..

[2] Tulyathan V., Leeharatanaluk B. (2007). Change in quality of rice (*Oryza sativa* L.) cv. Khao Dawk Mali 105 during storage. J. Food Biochem..

[3] Siangliw J., Jongdee B., Pantuwan G., Toojinda T. (2007). Developing KDML105 backcross introgression lines using marker-assisted selection for QTLs associated with drought tolerance in rice. Sci. Asia.

[4] Siddique M.R.B., Hamid A., Islam M.S. (2001). Drought stress effects on water relations of wheat. Bot. Bull. Acad. Sin..

[5] Hussain M., Malik M.A., Farooq M., Ashraf M.Y., Cheema M.A. (2008). Improving drought tolerance by exogenous application of glycine-betaine and salicylic acid in sunflower. J. Agron. Crop Sci..

[6] Cornic G., Massacci A., Baker N.R. (1996). Leaf photosynthesis under drought. Photosynthesis and the Environment.

[7] Anjum F., Yaseen M., Rasul E., Wahid A., Anjum S. (2003). Water stress in barley (*Hordeum valgare* L.). I. effect on chemical composition and chlorophyll content. Pak. J. Agron. Sci..

[8] Du Y.C., Kawamitsu Y., Nose A., Hiyane S., Murayama S., Wasano K., Uchida Y. (1996). Effect on water stress on carbon exchange rate and activities of photosynthetic enzymes in leaves of sugarcane (*Saccharum* sp.). Aust. J. Plant Physiol..

[9] Farooq M., Wahid A., Kobayashi N., Fujita D., Basra S.M.A. (2009). Plant drought stress: Effects, mechanisms and management. Agron. Sustain. Dev..

[10] Zargar S.M., Gupta N., Nazir M., Mahajan R., Malik F.A., Sofi N.R., Shikari A.B., Salgotra R.K. (2017). Impact of drought on photosynthesis: Molecular perspective. Plant Gene.

[11] Kanjoo V., Punyawaew K., Siangliw J.L., Jearakongman S., Vanavichit A., Toojinda T. (2012). Evaluation of agronomic traits in chromosome segment substitution lines of KDML105 containing drought tolerance QTL under drought stress. Rice Sci..

[12] Punchkhon C., Kasetranunt W., Kositsup B., Siengliw J., Chadchawan S. Evaluation of drought tolerance ability at seedling stage of CSSL rice populations with drought tolerant genes on chromosome 8. Proceedings of the 9th Botanical Conference of Thailand.

[13] Vajrabhaya M., Vajrabhaya T., Bajaj Y.P.S. (1991). Somaclonal variation of salt tolerance in rice. Biotechnology Agriculture Forestry.

[14] Pongprayoon W., Roytrakul S., Pichayangkura R., Chadchawan S. (2013). The role of hydrogen peroxide in chitosan-induced resistance to osmotic stress in rice (*Oryza sativa* L.). Plant Growth Regul..

[15] Chintakovid N., Maipoka M., Phaonakrop N., Mickelbart M.V., Roytrakul S., Chadchawan S. (2017). Proteomic analysis of drought-responsive proteins in rice reveals photosynthesis-related adaptations to drought stress. Acta Physiol. Plant..

[16] Missirian V., Comai L., Filkov V. (2011). Statistical mutation calling from sequenced overlapping DNA pools in TILLING experiments. BMC Bioinf..

[17] Ouyang S., Zhu L., Hamilton J., Lin H., Campbell M., Childs K., Thibaud-Nissen F., Malek R.L., Lee Y., Zheng L. (2007). The TIGR rice genome annotation resource: Improvements and new features. Nucleic Acids Res..

[18] Kawahara Y., de la Bastide M., Hamilton J.P., Kanamori H., McCombie W.R., Ouyang S., Schwartz D.C., Tanaka T., Zhou S., Childs K.L. (2013). Improvement of the *Oryza sativa* Nipponbare reference genome using next generation sequence and optical map data. Rice.

[19] Cao P., Jung K., Choi D., Hwang D., Zhu J., Ronald P. (2012). The rice oligonucleotide array database: An atlas of rice gene expression. Rice.

[20] Xia L., Zou D., Sang J., Xu X.J., Yin H.Y., Li M.W., Wu S.Y., Hu S.N., Hao L.L., Zhang Z. (2017). Rice Expression Database (RED): An integrated RNA-Seq-derived gene expression database for rice. J. Genet. Genom..

[21] Lamesch P., Berardini T.Z., Li D., Swarbreck D., Wilks C., Sasidharan R., Muller R., Dreher K., Alexander D.L., Garcia-Hernandez M. (2012). The arabidopsis information resource (TAIR): Improved gene annotation and new tools. Nucleic Acids Res..

[22] De Diego N., Fürst T., Humplik J., Ugena L., Podlešáková K., Spíchal L. (2017). An automated method for high-throughput screening of Arabidopsis rosette growth in multi-well plates and its validation stress condition. Front. Plan Sci..

[23] Yonemaru J., Yamamoto T., Fukuoka S., Uga Y., Hori K., Yano M. (2010). Q-TARO:QTL Annotation rice online database. Rice.

[24] Tu C.J., Schuenemann D., Hoffman N.E. (1999). Chloroplast FtsY, chloroplast signal recognition particle, and GTP are required to reconstitute the soluble phase of light-harvesting chlorophyll protein transport into thylakoid membranes. J. Biochem. Chem..

[25] Rumeau D., Bécuwe-Linka N., Beyly A., Louwagie M., Garin J., Peltier G. (2005). New subunit NDH-M, -N, -O, encoded by nuclear genes, are essential for plastid Ndh complex functioning in higher plants. Plant Cell.

[26] Brooks M.D., Sylak-Glassman E.J., Fleming G.R., Niyogi K.K. (2013). A thioredoxin-like/β-propeller protein maintains the efficiency of light harvesting in *Arabidopsis*. Proc. Natl. Acad. Sci. USA.

[27] Adamiec M., Gibasiewicz K., Luciński R., Giera W., Chełminiak P., Szewczyk S., Sipińska W., Grondelle R., Jackowski G. (2015). Excitation energy transfer and charge separation are affected in *Arabidopsis thaliana* mutants lacking light-harvesting chlorophyll *a/b* binding protein Lhcb3. J Photochem Photobiol. B Biol..

[28] Guan Z., Wang W., Yu X., Lin W., Maio Y. (2018). Comparative proteomic analysis of coregulation of CIPK14 and WHIRLY1/3 mediated pale yellowing of leaves in *Arabidopsis*. Int. J. Mol. Sci..

[29] Hertle A.P., Blunder T., Wunder T., Pesaresi P., Pribil M., Armbruster U., Leister D. (2013). PGRL1 is the elusive ferredoxin-plastoquinone reductase in photosynthetic cyclic electron flow. Mol. Cell.

[30] Hey D., Grimm B. (2018). ONE-HELIX PROTEIN2 (OHP2) is required for the stability of OHP1 and assembly factor HCF244 and is functionally linked to PSII biogenesis. Plant Physiol..

[31] Li Y., Liu B., Zhang J., Kong F., Zhang L., Meng H., Li W., Rochaix J.D., Li D., Peng L. (2019). OHP1, OHP2, and HCF244 form a transient functional complex with the photosystem II reaction center. Plant Physiol..

[32] Chotewutmontri P., Williams-Carrier R., Barkan A. (2020). Exploring the link between photosystem II assembly and translation of the chloroplast *psbA* mRNA. Plants.

[33] Motohashi R., Yamazaki T., Myouga F., Ito T., Ito K., Satou M., Kobayashi M., Nagata N., Yoshida S., Nagashima A. (2007). Chloroplast ribosome release factor 1 (AtcpRF1) is essential for chloroplast development. Plant Mol. Biol..

[34] Tezara W., Mitchell V.J., Driscoll S.D., Lawlor D.W. (1999). Water stress inhibits plant photosynthesis by decreaseing coupling factor and ATP. Nature.

[35] Teulat B., Monneveux P., Wery J., Borries C., Souyris I., Charrier A., This D. (1997). Relationships between relative water content and growth parameters under water stress in barley: A QTL study. New Phytol..

[36] González L., González-Vilar M. (2001). Determination of relative water content. Handbook of Plant Ecophysiology Techniques.

[37] Nikkanen L., Toivola J., Trotta A., Diaz M.G., Tikkanen M., Aro E., Rintamäki E. (2018). Regulation of cyclic electron flow by chloroplast NADPH-dependent thioredoxin system. Plant Direct.

[38] Damkjaer J.T., Kereïche S., Johnson M., Kovacs L., Kiss A.Z., Boekema E.J., Ruban A.V., Horton P., Jansson S. (2009). The photosystem II light-harvesting protein Lhcb3 affects the macrostructure of photosystem II and the rate of state transitions in *Arabidopsis*. Plant Cell.

[39] Wang L., Ouyang M., Li Q., Zou M., Gou J., Ma J., Lu C., Zhang L. (2010). The *Arabidopsis* chloroplast ribosome recycling factor is essential for embryogenesis and chloroplast biogenesis. Plant Mol. Biol..

[40] DalCorso G., Pesaresi P., Masiero S., Aseeva E., Schünemann D., Finazzi G., Joliot P., Barbato R., Leister D. (2008). A complex containing PGRL1 and PGRL5 is involved in the switch between linear and cyclic electron flow in *Arabidopsis*. Cell.

[41] Suorsa M., Rossi F., Tadini L., Labs M., Colombo M., Jahns P., Kater M.M., Leister D., Finazzi G., Aro E. (2016). PGRL5-PGRL1-dependent cyclic electron transport modulates linear electron transport rate in *Arabidopsis thaliana*. Mol. Plant.

[42] Wang F., Yan J., Ahammed G.J., Wang X., Bu X., Xiang H., Li Y., Lu J., Liu Y., Qi H. (2020). PGR5/PGRL1 and NDH mediate far-red light-induced photoprotection in response to chilling stress in tomato. Front. Plant Sci..

[43] Johnson X., Wostrikoff K., Finazzi G., Kuras R., Schwarz C., Bujaldon S., Nickelsen J., Stern D.B., Wollman F., Vallon O. (2010). MRL1, a conserved pentatricopeptide repeat protein, is required for stabilization of *rbcL* mRNA in *Chlamydomas* and *Arabidopsis*. Plant Cell.

